# The Effects of Dietary Supplementation with 25-Hydroxyvitamin D3 on the Antioxidant Capacity and Inflammatory Responses of *Pelteobagrus fulvidraco*

**DOI:** 10.3390/biology14080967

**Published:** 2025-08-01

**Authors:** Yi Liu, Jiang Xie, Qingchao Shi, Quan Gong, Chuanjie Qin

**Affiliations:** 1 Key Laboratory of Sichuan Province for Fishes Conservation and Utilization in the Upper Reaches of the Yangtze River, Neijiang Normal University, Neijiang 641000, China; liuyi_shz@163.com (Y.L.); xiejianggz@163.com (J.X.); gaoyisqc@126.com (Q.S.); 2 Special Agricultural Resources in Tuojiang River Basin Sharing and Service Platform of Sichuan Province, Neijiang Normal University, Neijiang 641000, China; 3 College of Life Sciences, Neijiang Normal University, Neijiang 641100, China; 4 Fishery Institute, Sichuan Academy of Agricultural Sciences, Chengdu 611731, China; admiral671@163.com

**Keywords:** metabolome, transcriptome, yellow catfish, 25(OH)D3, antioxidant, inflammatory

## Abstract

Based on the limited hepatic hydroxylation efficiency of dietary VD3 in teleosts and the superior bioavailability of its metabolite, 25(OH)D3, this study investigated the regulatory mechanisms of dietary 25(OH)D3 supplementation in yellow catfish—an economically significant species lacking prior nutritional data on this metabolite. A total of 360 fish were divided into three groups—control (basal diet), VD3 (2500 IU/kg VD3), and 25(OH)D3 (2500 IU/kg 25(OH)D3)—and fed for 8 weeks. Compared to the control, both supplemented groups showed elevated superoxide dismutase (SOD), total antioxidant capacity (T-AOC), catalase (CAT), and transforming growth factor-β (*TGF-β*) activities, alongside reduced malondialdehyde (MDA), interleukin-1β (*IL-1β*), and tumor necrosis factor-α (*TNF-α*) levels. The 25(OH)D3 group exhibited higher T-AOC and CAT activities and lower *TNF-α* than the VD3 group. Metabolomic and transcriptomic analyses identified 65 differentially expressed metabolites (DEMs) and 3515 differentially expressed genes (DEGs). Enrichment analysis indicated that the DEMs (e.g., indole compounds, organic acids, aldosterone, L-kynurenine) and DEGs (*pgd*, *mthfr*, *nsdhl*, *nox5*, *prdx2*, *mpx*, *itih2*, *itih3*, *eprs1*) that were highly and significantly expressed in the 25(OH)D3 group were primarily associated with antioxidant defense and inflammatory responses. Dietary 25(OH)D3 was more effective than VD3 in promoting antioxidant capacity and modulating inflammation in yellow catfish.

## Simple Summary

25-Hydroxyvitamin D3 (25(OH)D3), the active metabolite of vitamin D3 (VD3) in vivo, exhibits greater biological activity and efficiency than VD3. This study investigated the effects of substituting VD3 with 25(OH)D3 in feed on intestinal antioxidant capacity and inflammatory responses in yellow catfish (*Pelteobagrus fulvidraco*). The results demonstrated that dietary 25(OH)D3 significantly enhanced the expression of antioxidant enzymes, anti-inflammatory factors, and key metabolites (organic acids and indole compounds), while reducing lipid peroxidation products and pro-inflammatory factors, compared to VD3. Transcriptomic analysis revealed the up-regulation of genes involved in antioxidant defense, organic acid metabolism, lipid metabolism, and terpenoid backbone biosynthesis in the 25(OH)D3 group. The multi-omics convergence supports that 25(OH)D3 outperforms VD3 in promoting antioxidant capacity and modulating inflammation in yellow catfish.

## 1. Introduction

**Figure 1:**
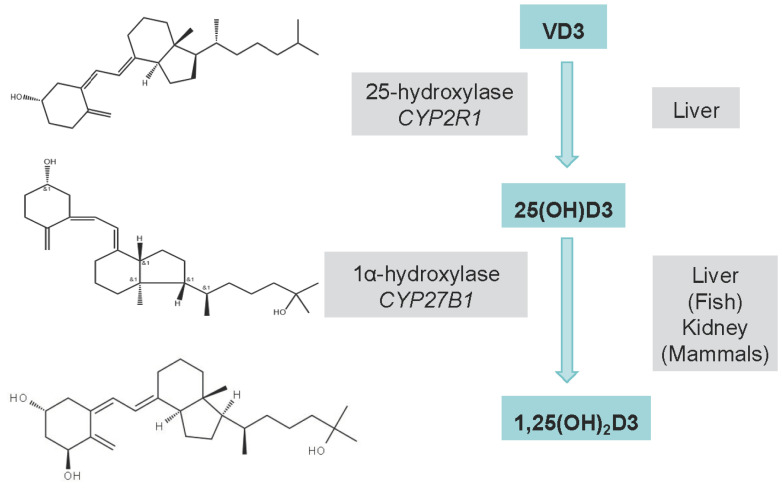
Comparative metabolic pathways of vitamin D3 in fish and mammals. “&1” represents a wedge bond, used to depict the three-dimensional structure of a molecule. The -H and -CH groups connected by “&1” are positioned in front of the plane of the paper, indicating the stereochemical configuration of the molecule at this location. VD3 undergoes two-step activation: (1) hepatic 25-hydroxylation by *CYP2R1* produces 25(OH)D3, followed by (2) renal 1α-hydroxylation by *CYP27B1* to form 1,25(OH)D3 [1]. Key species differences are highlighted: in fish, both hydroxylation steps occur primarily in the liver, whereas mammals distribute these reactions between liver (25-hydroxylation) and kidneys (1α-hydroxylation) [2].

Despite the critical role of vitamin D in vertebrate physiology, the metabolic divergence of vitamin D3 (VD3) in teleosts—where inefficient hepatic hydroxylation limits bioactivation—creates a fundamental nutritional challenge for aquaculture, necessitating targeted supplementation strategies with its pre-hydroxylated metabolite 25-hydroxyvitamin D3 (25(OH)D3) in economically vital species like yellow catfish. VD3 is sequentially hydroxylated by *CYP2R1* (liver) and *CYP27B1* (kidney) to form 25(OH)D3 and active 1,25(OH)_2_D3, respectively (Figure 1) [1]. Notably, VD3 metabolism in aquatic species diverges from terrestrial vertebrates: hydroxylation occurs primarily in the liver of fish versus the kidneys of mammals [2]. While dietary VD3 accumulates dose-dependently in plasma, liver, and muscle, its conversion to 25(OH)D3 is limited. This inefficiency may explain why supra-nutritional VD3 fails to enhance growth and can cause renal toxicity [3]. 25(OH)D3, the circulating metabolite of VD3, is more efficiently absorbed and exhibits greater bioactivity than VD3, with a relative potency of 1.1–4.0 times at 3.125 μg/kg [4]. By bypassing hepatic 25-hydroxylation, dietary 25(OH)D3 shortens the metabolic pathway, offering enhanced stability and fewer adverse effects than 1,25(OH)_2_D3 [5]. Studies in rainbow trout (*Oncorhynchus mykiss*) reveal poor in vivo conversion of VD3 to 25(OH)D3 [6]. Indoor-reared fish lack cutaneous VD3 synthesis, further limiting conventional VD3 efficacy. Direct 25(OH)D3 supplementation circumvents hepatic hydroxylation, yielding ≥2-fold higher plasma 25(OH)D3 levels and superior bioavailability [7]. Recent work shows that calcifediol 25(OH)D3 at thrice the NRC requirements improves zootechnical performance in Atlantic salmon [8]. Its enhanced water solubility ensures efficient absorption under lipid malabsorption conditions (e.g., plant-based diets), making it ideal for aquafeeds with >73% plant-derived ingredients [9].

Yellow catfish (*Pelteobagrus fulvidraco*) is a major economic freshwater species in China, with expanding markets across East and South Asia [10]. Rapid intensification of farming necessitates optimized nutrition. Previous studies indicate that nutrients (e.g., copper, protein, zinc, and cadmium) influence growth [11], though VD3 supplementation showed no significant effects [12]. No data exist on 25(OH)D3 in yellow catfish. Metabolomics and transcriptomics are powerful tools for evaluating feed additive impacts on fish health [13,14]. This study investigated the regulatory mechanisms of dietary 25(OH)D3 in yellow catfish using these approaches, providing a foundation for its rational application in aquaculture.

## 2. Materials and Methods

### 2.1. Sample Collection

The feed protein source was composed of fish meal, soybean meal, wheat flour, and chicken meal, and the fat source was soybean oil. The above materials were provided by Puxiang Agricultural Science and Technology Co., Ltd. (Neijiang, China). The VD3 and 25(OH)D3 were provided by Hechen Agricultural Science and Technology Co., Ltd. (Meishan, China), and Pilot Biotechnology Co., Ltd. (Suzhou, China), respectively. The raw materials were crushed, sieved, and mixed to form three experimental diets: the control group (the diet contained no VD3 supplementation of any form, and VD3 was excluded during the vitamin premix formulation), experimental group I (2500 IU/kg VD3), and experimental group II (2500 IU/kg 25(OH)D3), respectively. The supplementation levels for yellow catfish were determined as 2500 IU/kg for both VD3 and 25(OH)D3, based on (a) the optimal supplementation level of VD3 for other fish species (VD3: 1994.8–2321.8 IU/kg in *Ctenopharyngodon idella* [15]; 2000 IU/kg in *Epinephelus coioides* [16]) and (b) the growth-optimal 25(OH)D3 level for *Litopenaeus vannamei* was 2615 IU/kg [17]. The experimental feed was dried in the oven at 55 °C, divided into marked sealed bags, and stored in the refrigerator at −20 °C. About 20 g of experimental feed was taken from each group for nutrient composition analysis. Table S1 shows the composition and nutrient level of experimental feed. The protein–lipid ratio aligns with the optimal ranges in yellow catfish [18]. Ash content ensures adequate mineral balance for bone health [12]. The experimental fish were obtained from a Neijiang City hatchery and temporarily cultured at Jiazhi Fish Breeding Farm’s pond, located in the Central District of Neijiang City. Fish were confirmed healthy via (1) visual/behavioral screening, (2) random pathogen testing, and (3) 2-week acclimation with <2% mortality. A total of 360 healthy yellow catfish juvenile (three months old; body length: 5.4 ± 0.28 cm; body weight: 5.01 ± 0.89 g) were randomly divided into outdoor net cages (with dimensions of 0.5 m × 0.5 m × 1.5 m, length/width/height), with 40 fish per cage. Then, the 9 fish cages were randomly divided into 3 groups. The breeding environment of this experiment was carried out under natural light outdoors. Daily feed ration was adjusted to 4–6% of total biomass per cage based on observed consumption patterns. Fish were fed twice daily (07:00 and 19:00) throughout the 8-week culture period. Water quality parameters during the trial were as follows: temperature: 25–31 °C; dissolved oxygen: 7.06–7.32 mg/L; pH 7.9–8.3; and ammonia nitrogen: ≤ 0.30 mg/L. A 100% survival rate was observed across all experimental groups upon completion of the culture period. After the aquaculture cycle, 20 fish were randomly sampled from each replicate. Under sterile conditions, both intestinal and head kidney tissues were harvested, washed with sterile physiological saline (0.9% sodium chloride), and immediately placed in RNase/DNase-free microcentrifuge tubes. Then, we cut the intestines with scissors and gently scraped the contents with tweezers to avoid scraping the intestinal lining mucosa as much as possible. Samples were flash-frozen in liquid nitrogen and stored at −80 °C.

### 2.2. Detection of Intestinal Antioxidase Activities

Intestinal tissues were homogenized in ice-cold saline (1:9 *w*/*v*, 3–5 min) and centrifuged (70× *g*, 10 min, 4 °C), and the supernatants analyzed for superoxide dismutase (SOD), total antioxidant capacity (T-AOC), catalase (CAT), and malondialdehyde (MDA) using commercial kits (Jiancheng Bioengineering Institute, Nanjing, China). Data (mean ± SD) were analyzed by one-way ANOVA (SPSS 21.0), with post hoc Duncan’s tests (*p* < 0.05).

### 2.3. Detection of Intestinal Inflammatory Cytokine Genes

Total RNA was extracted with TRIzol reagent (Thermo Fisher Scientific, #15596026; Waltham, MA, USA), quantified using NanoDrop 2000 (Thermo Fisher Scientific, Wilmington, DE, USA), and quality-checked (1% agarose gel). Qualified RNA was reverse-transcribed using PrimeScript RT Master Mix (Takara Bio, #RR036A; Kusatsu, Shiga, Japan). Gene expression of interleukin-1β (*IL-1β*), interleukin-8 (*IL-8*), tumor necrosis factor-α (*TNF-α*), and transforming growth factor-β (*TGF-β*) was quantified by qRT-PCR using ABI 7500 system (Applied Biosystems, Foster City, CA, USA) with SYBR Premix Ex Taq™ (Takara Bio, #RR420A; Kusatsu, Shiga, Japan), using β-actin as reference. The protocol: 95 °C for 3 min; 40 cycles of 95 °C (5 s), 60 °C (1 min), and 72 °C (30 s). All reactions were run in triplicate, with relative expression calculated by 2^−ΔΔCt^ [19]. Primers (designed by Primer Premier v5.0) were synthesized by TSINGKE Biotech (Beijing, China). Primer sequences and NCBI accession numbers for β-actin and target genes are listed in Supplementary Table S2.

### 2.4. Metabolites Extraction

Exactly 100 mg of intestinal contents was weighed using a Mettler Toledo XSR microbalance (±0.1 mg precision). Three independent replicates were established for each experimental group. Intestinal contents were cryogenically ground in liquid nitrogen then homogenized in prechilled 80% methanol (vortex-mixed for 30 s using Genie 2). After ice incubation (5 min) and centrifugation (300× *g*, 5 min for debubbling), samples underwent two-stage extraction: (1) primary centrifugation (15,000× *g*, 20 min, 4 °C); (2) dilution to 53% methanol (LC-MS grade water) and secondary centrifugation (same parameters). Final supernatants were analyzed by LC-MS/MS [20].

### 2.5. UHPLC-MS/MS Analysis

UHPLC-MS/MS analyses were performed by Novogene Co., Ltd. (Beijing, China) using a ThermoFisher Vanquish UHPLC system coupled to an Orbitrap Q Exactive™ HF-X mass spectrometer. Separation was achieved on a Hypersil Gold column (100 mm × 2.1 mm, 1.9 μm) with a 0.2 mL/min flow rate and 17-minute linear gradient. Mobile phases consisted of (A) 0.1% formic acid in water (positive mode) or 5 mM ammonium acetate (pH 9.0, negative mode) and (B) methanol for both modes. The gradient program was 2% B (0–1.5 min), 2–85% B (1.5–4.5 min), and 85–100% B (4.5–14.5 min), returning to 2% B at 14.6 min and holding until 17 min. MS parameters included a 3.5 kV spray voltage, 320 °C capillary temperature, 35 psi sheath gas, 10 L/min auxiliary gas, 60 S-lens RF level, and 350 °C auxiliary gas heater temperature.

### 2.6. Data Processing and Metabolite Identification

Raw UHPLC-MS/MS data were processed using Compound Discoverer 3.1 (Thermo Fisher Scientific, Wilmington, DE, USA) with the following parameters: 0.2 min retention time tolerance, 5 ppm mass tolerance, 30% intensity tolerance, S/N ratio ≥ 3, and minimum intensity threshold. After total spectral intensity normalization, molecular formulas were predicted using additive ions, molecular ion peaks, and fragment ions. Metabolite identification was performed by matching peaks against mzCloud, mzVault, and MassList databases. Statistical analysis employed R v3.4.3, Python v2.7.6, and CentOS v6.6, with non-normally distributed data standardized using sample raw value/(∑sample metabolites/∑QC1 metabolites). Metabolites showing >30% coefficients of variation (CVs) in QC samples were excluded [21], yielding final identification and relative quantification results.

### 2.7. Data Analysis

Metabolites were annotated using the KEGG database, with differential metabolites identified by univariate analysis (*t*-test; *p* < 0.05), VIP (Variable Importance in Projection) scores >1, and fold change thresholds (>1.2 or <0.833) [22,23,24]. Data visualization included the following: (1) volcano plots (ggplot2) based on log2(FoldChange) versus −log10(*p*-value); and (2) clustering heatmaps (Pheatmap) of z-score normalized intensity areas. Metabolite correlations were analyzed using Pearson’s method (cor() function), with significance determined by cor.mtest() (*p* < 0.05) and visualized via corrplot. Pathway enrichment analysis considered pathways significant when both *p* < 0.05 and the ratio x/n > y/N, utilizing KEGG’s comprehensive biological systems database (www.genome.jp/kegg, accessed on 7 March 2025) for functional interpretation.

### 2.8. Transcriptome Sequencing and Quality Control

The head kidney tissues were used for transcriptome sequencing, with 5 replicates in each group. Novogene Co., Ltd. (Beijing, China) performed RNA extraction (RNeasy Mini Kit, QIAGEN, #74104; Hilden, Germany), library preparation (300–500 bp insert size) [25], and paired-end sequencing (Illumina HiSeq 2000 platform, Illumina, San Diego, CA, USA), with stringent quality control applied to raw sequencing data. The raw data were processed through a standardized quality control pipeline: (1) adapter sequences were trimmed using a custom perl script; (2) reads containing >10% ambiguous bases (N) or with >50% bases with Phred quality scores ≤ 20 were discarded. This stringent filtering process generated high-quality clean data for downstream analyses.

### 2.9. Reads Mapping and Quantification of Gene Expression Level

The yellow catfish reference genome was downloaded from the NCBI (Genebank entry number: GCA_022655615.1). The reference genome was indexed and paired-end reads were aligned using Hisat2 v2.0.5 [26], selected for its splice-aware alignment capability that utilizes gene annotation to improve mapping accuracy. Gene-level read counts were obtained using featureCounts v1.5.0-p3 [27], followed by FPKM calculation to normalize expression levels by gene length and sequencing depth [28].

### 2.10. Differential Expression Analysis and Functional Enrichment Analysis

Differential expression between conditions was analyzed using DESeq2 v1.20.0 [29], which applies negative binomial distribution-based statistics to RNA-seq data. Significant differentially expressed genes (DEGs) were identified using an adjusted *p*-value threshold of ≤0.05 (Benjamini-Hochberg FDR correction) [30]. The clusterProfiler R package v4.0.0 was used to perform both GO enrichment analysis (with gene length bias correction) and KEGG pathway enrichment analysis of DEGs, with terms/pathways showing corrected *p*-values < 0.05 considered statistically significant.

## 3. Results

### 3.1. Intestinal Antioxidant Enzyme Activities

Both VD3 and 25(OH)D3 supplementation significantly enhanced intestinal SOD, T-AOC, and CAT activities (*p* < 0.05) while reducing MDA levels compared to controls. Notably, 25(OH)D3 exhibited superior efficacy to VD3 in elevating T-AOC and CAT (*p* < 0.05), though no intergroup differences were observed for SOD and MAD (Table 1).

### 3.2. Intestinal Inflammatory Factors

**Figure 2:**
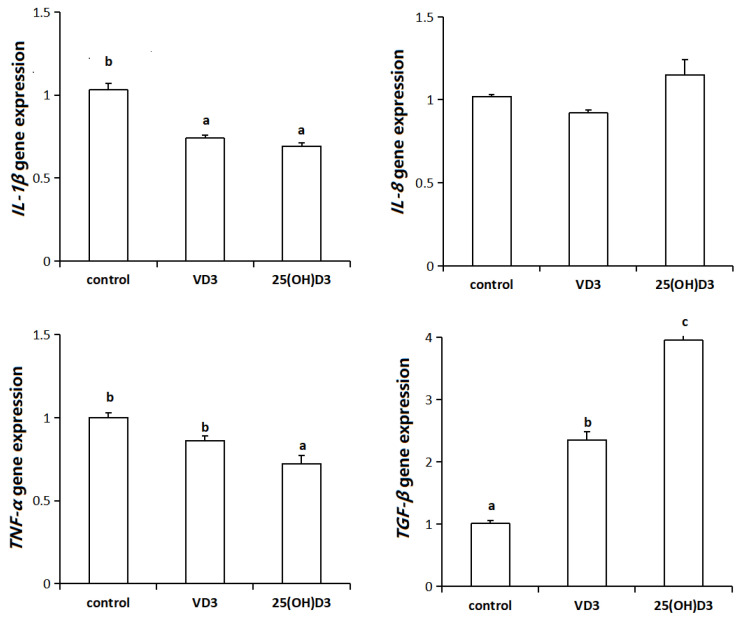
Effects of 25(OH)D3 on intestinal inflammatory factors. Different superscript letters (a, b, c) between columns indicate a significant difference (*p* < 0.05).

Compared with the control group, IL-1β levels were significantly decreased in both the VD3 and 25(OH)D3 groups (*p* < 0.05). In contrast, TGF-β levels were significantly increased in both the VD3 and 25(OH)D3 groups (*p* < 0.05). TNF-α levels in the 25(OH)D3 group were significantly lower than those in both the control and VD3 groups (*p* < 0.05). No significant changes were observed in IL-8 levels across all experimental groups (Figure 2).

### 3.3. Metabolites Obtained Based on LC-MS Detection

**Figure 3:**
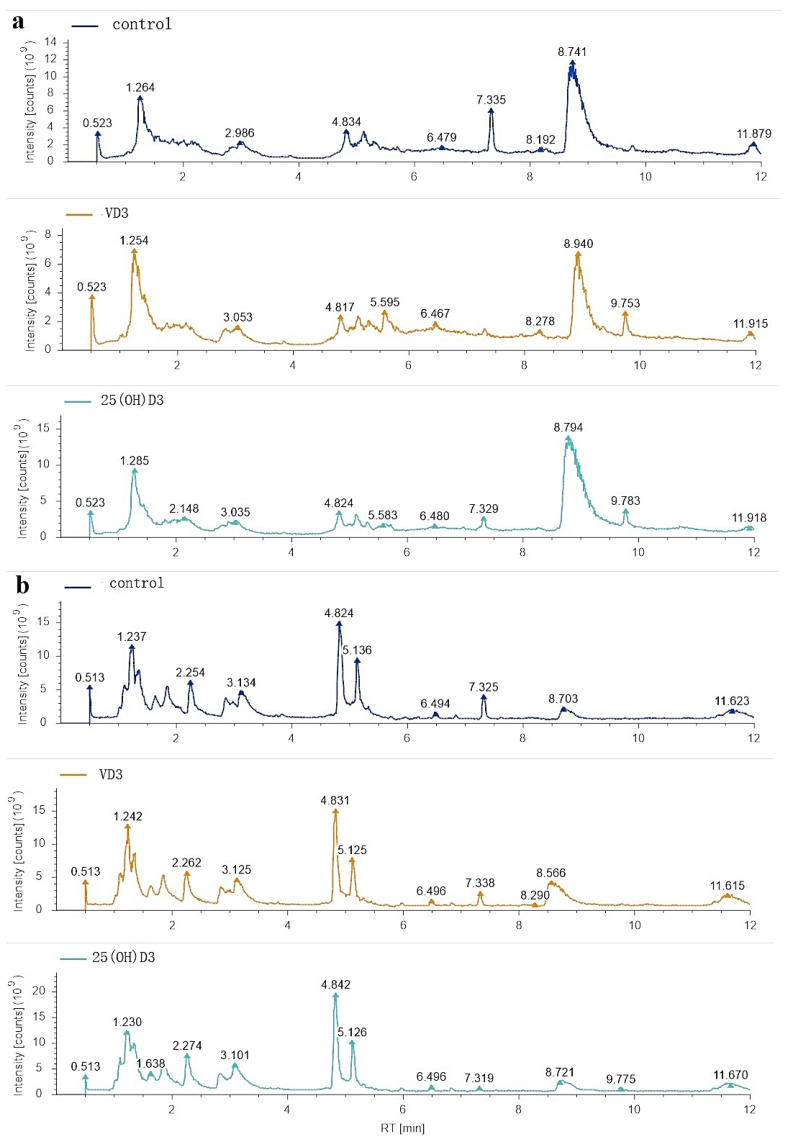
Total ion chromatogram (TIC) of the control, VD3 and 25(OH)D3 groups: (**a**) positive ion mode (POS); (**b**) negative ion mode (NEG).

Three experimental groups were set up in the metabolic group with three replicates per group: control group (A), VD3 group (B), 25(OH)D3 group (C). The total ion chromatogram of metabolites showed that the chromatographic peak separation of different components was better in the positive ion mode (POS) (Figure 3a) and negative ion mode (NEG) (Figure 3b). Intuitive comparison showed that there were significant differences in some peak spectra among the three comparison groups. A total of 549 POS metabolites and 402 NEG metabolites were identified from nine samples. The chemical classification of identified metabolites was statistically analyzed. There were 11 categories: lipids and lipid-like molecules (29.99%); organic acids and derivatives (26.25%); organoheterocyclic compounds (14.33%); nucleosides, nucleotides, and analogues (11.51%); benzenoids (7.11%); organic oxygen compounds (5.39%); phenylpropanoids and polyketides (2.52%); organic nitrogen compounds (1.89%); alkaloids and derivatives (0.43%); none (0.43%); and hydrocarbons (0.15%).

### 3.4. Identification and Analysis of Differential Metabolites

**Figure 4:**
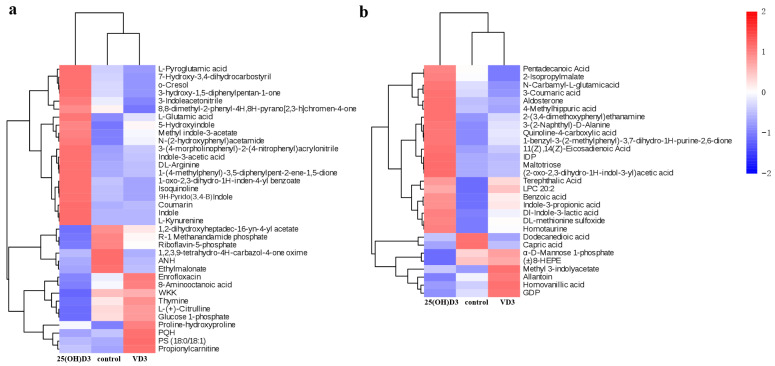
Clustering heat map of differential metabolites. The horizontal direction represents the clustering of metabolites, and the vertical direction represents the sample type. The shorter the clustering branch, the higher the similarity. (**a**) Positive ion mode (POS); (**b**) negative ion mode (NEG).

There was a total of 65 (36 POS metabolites (Figure 4a) and 29 NEG metabolites (Figure 4b)) differentially expressed metabolites (DEMs) were obtained from pairwise comparisons of groups. Six POS metabolites (one up-regulated and five down-regulated) and four NEG metabolites (two up-regulated and two down-regulated) were differentially expressed between the control group and the VD3 group (Table S3). A total of 18 POS metabolites (5 up-regulated and 13 down-regulated) and 19 NEG metabolites (3 up-regulated and 16 down-regulated) were differentially expressed between the control group and the 25(OH)D3 group (Table S4). A total of 21 POS metabolites (10 up-regulated and 11 down-regulated) and 11 NEG metabolites (5 up-regulated and 6 down-regulated) were differentially expressed between the VD3 group and the 25(OH)D3 group (Table S5). Hierarchical cluster analysis of DEMs revealed that the control group and VD3 cluster together, indicating a smaller difference between them compared to the difference between the control and 25(OH)D3 groups (Figure 4). Dietary addition of 25(OH)D3 has a more significant effect on the metabolism of yellow catfish than VD3.

**Figure 5:**
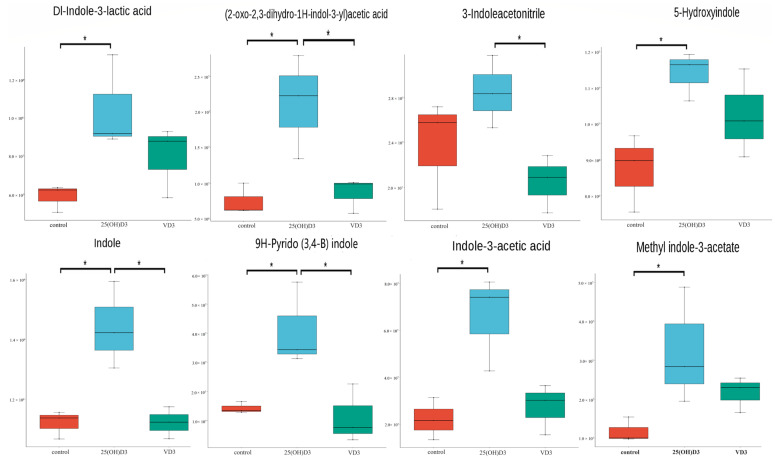
Expression levels of the indole compounds in the control, VD3, and 25(OH)D3 groups. Box plots with *p*-values of Dl-indole-3-lactic acid, (2-oxo-2,3-dihydro-1H-indol-3-yl) acetic acid, 3-indoleacetonitrile, 5-hydroxyindole, indole, 9H-pyrido (3,4-B) indole, indole-3-acetic acid, and methyl indole-3-acetate. Data were analyzed by one-way ANOVA followed by Duncan’s multiple comparison test, with statistical significance set at *p* < 0.05. “*” indicates a significant difference between groups (*p* < 0.05).

The common metabolites with significant differences in the three comparison groups were extracted, and the results indicate that most of the metabolites whose quantitative values in the 25(OH)D3 group were significantly higher than those in the control group and the VD3 group were organic acids and derivatives, such as L-glutamic acid, L-pyroglutamic acid, terephthalic acid, benzoic acid, pentadecanoic acid, quinoline-4-carboxylic acid, N-carbamyl-L-glutamic acid, 3-coumaric acid, 11(Z),14(Z)-eicosadienoic acid, 4-methylhippuric acid, and (2-oxo-2,3-dihydro-1H-indol-3-yl) acetic acid (Figure 3). In addition, the differential metabolites contained many indole compounds (Dl-indole-3-lactic acid, (2-oxo-2,3-dihydro-1H-indol-3-yl) acetic acid, indole, indole-3-acetic acid, methyl indole-3-acetate, 3-inoleacetonitrile, 5-hydroxyindole, and 9H-pyrido (3,4-B) indole), and the quantitative values of these metabolites in the 25(OH)D3 group were higher than those in the control and the VD3 groups (Figure 5). Indole, a versatile heterocyclic compound, exhibits broad-spectrum bioactivity, including antioxidant, anti-inflammatory, antibacterial, antitumor, antiviral, immunomodulatory, and analgesic properties [31].

### 3.5. KEGG Enrichment Analyses of Differential Metabolites

**Figure 6:**
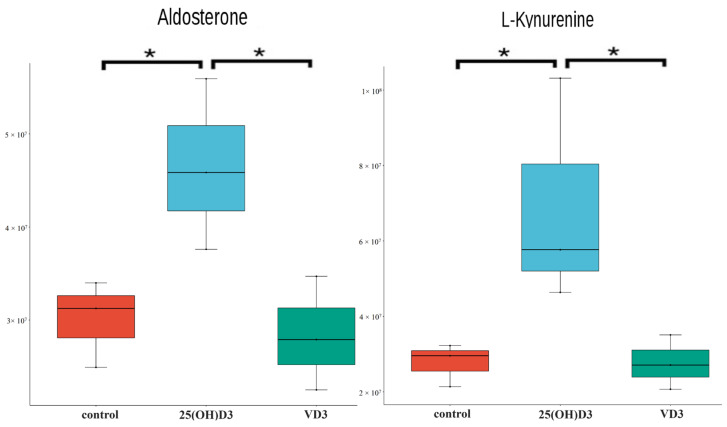
The expression levels of aldosterone and L-kynurenine in control, VD3, and 25(OH)D3 groups. Box plots with *p*-values of aldosterone and L-kynurenine. Figure 5 and Figure 6 employ consistent statistical significance testing methods. “*” indicates a significant difference between groups (*p* < 0.05).

KEGG enrichment analysis was performed based on the identified differential metabolites. There was no significant enrichment of the differential metabolism between the control group and the VD3 group. In NEG, aldosterone-regulated sodium reabsorption, aldosterone synthesis and secretion, and steroid hormone biosynthesis were enriched in both the control and 25(OH)D3 contrast groups (Table S6) and the VD3 and 25(OH)D3 contrast groups (Table S7). Aldosterone was the common metabolite enriched across all three pathways, with significantly higher levels in the 25(OH)D3 group compared to both the control and the VD3 groups (*p* < 0.05), while the control and the VD3 groups showed comparable levels (Figure 6). In POS, the co-enriched pathways include tryptophan metabolism, African trypanosomiasis, phenylalanine, tyrosine and tryptophan biosynthesis, protein digestion and absorption, and metabolic pathways. The metabolites enriched in these pathways were L-kynurenine, indole-3-acetic acid, and indole, all of which are metabolites of tryptophan and participate in antioxidant and inflammatory reactions.

### 3.6. Identification and Analysis of Differential Transcripts

Three groups were established with five replicates per group: control group (A1–A5); VD3 group (B1–B5); 25(OH)D3 group (C1–C5). A total of 95.18 G of raw data was sequenced, and 92.06 G of clean data was obtained after filtering, with an average of 5.69–6.49 G clean reads for each library. The GC content of each library was between 44.63% and 46.11%. The efficiency of sequencing was more than 90%, and the average base error rate was only 0.03%. In addition, Q20 ≥ 97% and Q30 ≥ 92%, indicating that the error rate of a single base was very low (Table 2). Finally, 34,599 transcripts were assembled in the 15 libraries. The correlation coefficients for the five replicates of the three groups were greater than 0.88, and the correlation of the samples within the group was greater than that of the samples between the groups. This ensured that more reliable results would be obtained from subsequent differential gene analysis.

**Figure 7:**
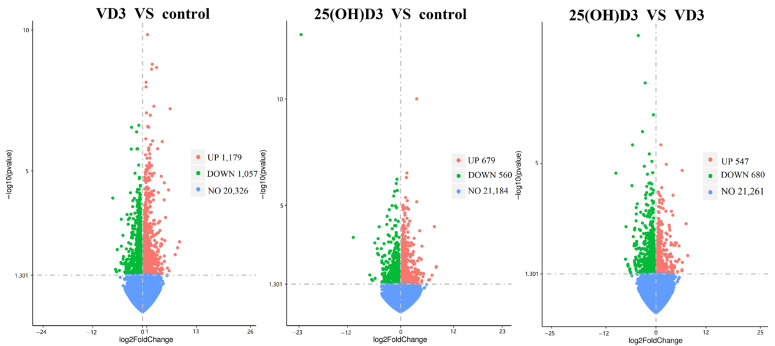
Differential gene volcano map. The horizontal coordinate represents the log2FoldChange value, the vertical coordinate is −log10padj, and the dotted line indicates the threshold line of the differential gene screening criteria.

Using DESeq2 (*p* value ≤ 0.05 |log2FoldChange| ≥ 0) as cutoffs, 3515 DEGs were obtained from the pairwise comparisons. A total of 2236 transcripts (1179 up-regulated and 1057 down-regulated) were differentially expressed between the control and VD3 groups. As many as 1239 transcripts (679 up-regulated and 560 down-regulated) were differentially expressed between the control and 25(OH)D3 groups. And a total of 1227 transcripts (547 up-regulated and 680 down-regulated) were differentially expressed between the VD3 and 25(OH)D3 groups (Figure 7).

### 3.7. Functional Enrichment Analyses of Differentially Transcripts

**Figure 8:**
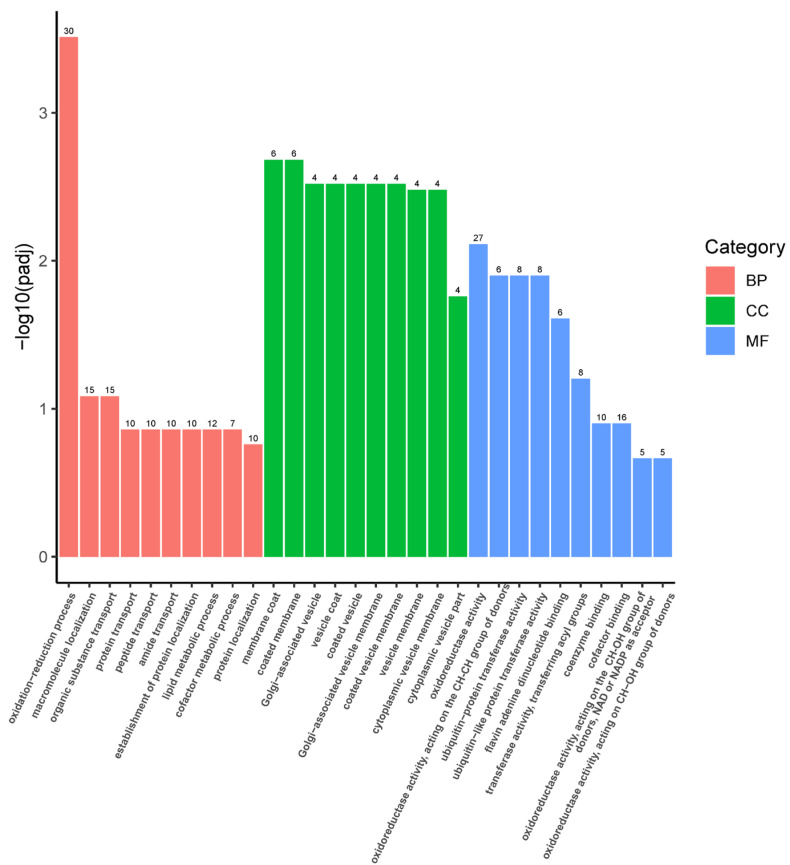
The up-regulated DEGs in the GO functional enrichment bar chart of the 25(OH)D3 and VD3 groups. The *x*-axis displays the GO terms, while the *y*-axis shows the enrichment significance (−log_10_[padj]). Color coding indicates functional categories. BP, biological processes; CC, cellular components; MF, molecular functions. The numbers on the column represent the number of differentially expressed genes annotated to the GO term.

There was no significant GO functional enrichment of down-regulated DEGs in the 25(OH)D3 vs. VD3 groups and 25(OH)D3 vs. control groups. The up-regulated DEGs GO functional significant enrichment in 25(OH)D3 and VD3 groups was associated with processes related to antioxidant defense, such as the following: the oxidation reduction process (GO:0055114) [32]; oxidoreductase activity (GO:0016491) [33]; oxidoreductase activity acting on the CH-CH group of donors (GO:0016627) [34]; oxidoreductase activity acting on the CH-OH group of donors, with NAD or NADP as the acceptors (GO:0016616) [35]; and oxidoreductase activity, acting on CH-OH group of donors (GO:0016614) [36] (Figure 8). The up-regulated DEGs *(mthfr*, *sqlea*, *nsdhl*, *impdh2*, *gcdhb*, *pdpr*, *acadvl*, *fasn*, *pgd*, *mpx*, *prdx2*, *pam*, *msmo1*, *etfdh*, *acadm*, *ldhba*, *acads*, *aldh1l2*, *hmgcra*, *acox1*, *gys2*, *hadhab*, *nox5*, and *aldh4a1*) involved in these processes are listed in Table 3.

Interestingly, the up-regulated DEGs in the 25(OH)D3 and control groups were enriched in the organic acid metabolic process (GO:0006082) [37]. This was consistent with the high expression of organic acids and derivatives in the 25(OH)D3 group in the results of the metabolome analysis. The up-regulated DEGs (*ehhadh*, *itih3*, *eprs1*, *itih2*, *hdc*, *dars1*, *mthfr*, *pklr*, and *yars2*) involved in the organic acid metabolic process are listed in the table (Table 4).

**Figure 9:**
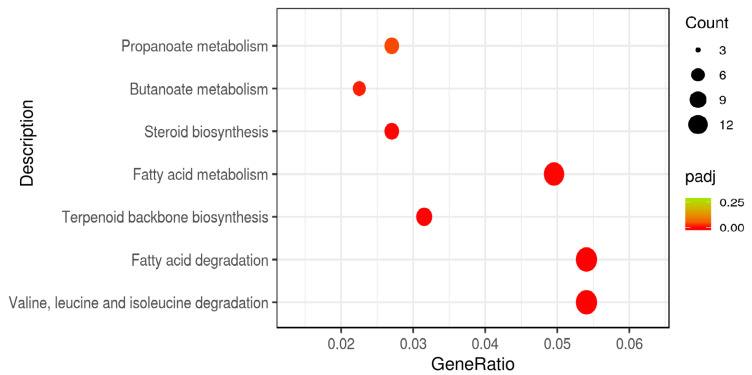
KEGG-rich distribution point plots of the up-regulated differentially expressed genes in the 25(OH)D3 and VD3 groups. The x-axis shows the enrichment ratio (DEGs in pathway/total DEGs), while the y-axis displays the KEGG pathways.

No significant pathway enrichment was observed for down-regulated DEGs in either the 25(OH)D3 vs. VD3 or the 25(OH)D3 vs. control comparisons. The up-regulated DEGs in the 25(OH)D3 and VD3 groups were significantly enriched to valine, leucine, and isoleucine degradation (ipu00280); fatty acid degradation (ipu00071); terpenoid backbone biosynthesis (ipu00900); fatty acid metabolism (ipu01212); steroid biosynthesis (ipu00100); butanoate metabolism (ipu00650); and propanoate metabolism signaling pathways (ipu00640) (Figure 9). Most of these pathways were related to the metabolism of lipids (steroids, fatty acids, butyrate, and propionate). In addition, seven genes were significantly up-regulated in the terpenoid backbone biosynthesis pathway, namely, *mvda*, *fdps*, *hmgcs1*, *idi1*, *nus1*, *acat2*, and hmgcra (Table 3). The expression of these genes in the 25(OH)D3 group was significantly higher than that in the VD3 group, suggesting that 25(OH)D3 supplementation can promote a synthesis of the terpenoids of yellow catfish to a greater degree than VD3 supplementation.

## 4. Discussion

### 4.1. The Addition of 25(OH)D3 Can Promote the Antioxidant Defense of Yellow Catfish

Our study reveals three key mechanisms by which 25(OH)D3 surpasses VD3 in enhancing antioxidant capacity. Enzyme activation: The significantly higher T-AOC and CAT levels (vs. VD3) suggest that 25(OH)D3 more effectively activates the Nrf2-Keap1 pathway, a master regulator of antioxidant enzymes [38]. Antioxidant enzymes, including SOD, PRDX, CAT, glutathione peroxidase (GSHPx), glutathione reductase (GR), and G6PD, are important substances in the body’s defense against oxidative stress [39]. CAT and SOD are among the most critical antioxidant enzymes in organisms, with CAT and SOD specifically acting on H_2_O_2_ and O_2_^−^, respectively [40]. T-AOC measures total antioxidant capacity, encompassing enzymes (CAT, SOD), vitamins (C, E), and carotenoids that collectively protect against ROS-induced oxidative damage [41]. Thus, T-AOC serves as a comprehensive indicator by which to evaluate the antioxidant efficacy of bioactive compounds. MDA levels typically reflect the extent of lipid peroxidation in vivo and indirectly indicate cellular damage [42]. Dietary supplementation with VD3 can enhance the levels of antioxidant enzymes (T-AOC, CAT, SOD) in the serum of largemouth bass and reduce the content of the lipid peroxidation product MDA [43]. Similarly, in this study, all measured antioxidant parameters (T-AOC, CAT, SOD) showed a significant upward trend in the VD3 and 25(OH)D3 groups, while MDA levels (a product of lipid peroxidation) exhibited an opposing pattern. The intestinal CAT, SOD, and T-AOC levels in yellow catfish supplemented with 25(OH)D3 were significantly elevated, demonstrating that 25(OH)D3 enhances the antioxidant system in yellow catfish, suggesting its beneficial role in intestinal health when added to feed.

NADPH generation: The up-regulation of *pgd*, *mthfr*, and *nsdhl* indicates enhanced NADPH production—a critical reductant for glutathione recycling. This aligns with murine models showing G6PD-mediated oxidative stress protection [44]. The steady-state level of NADPH is thought to determine the rate of damage caused by reactive oxygen species (ROS) [45]. In addition, some related NADPH-generating genes, namely, *mthfr* (*p* = 2 × 10^−6^), *nsdhl* (*p* = 0.003), and *nox5* (*p* = 0.045), and members of PRD family, namely, *prdx2* (*p* = 0.022) and *mpx* (*p* = 0.018), in the 25(OH)D3 group were also significantly higher than in the VD3 group. *Prdx2* can be used as an indicator of oxidative stress because it is a major red blood cell antioxidant, removing hydrogen peroxide formed by hemoglobin autooxidation endogenous [46]. *Prdx2* also reduces other peroxides, including lipids, uric acid, amino acids, protein hydroperoxides, and peroxynitrites [46]. In conclusion, 25(OH)D3-coordinated up-regulation of NADPH-generating enzymes (*pgd*, *mthfr*) and peroxide detoxifiers (*prdx2*, *mpx*) can enhance the antioxidant capacity of yellow catfish and protect cells from oxidative damage. Compared with VD3, 25(OH)D3 can promote the antioxidant defense ability of yellow catfish.

Indole-mediated protection: The increase in indole derivatives (vs. VD3) provides additional antioxidant capacity through radical scavenging [47]. Indole compounds exhibit potent antioxidant activity, preventing protein/lipid peroxidation in biological systems [31]. Indoles demonstrate potent oxygen free radical scavenging and are among the most effective acetylcholinesterase (AChE) inhibitors [48]. Among them, indole-3-propionic acid has good antioxidant activity [47]. Indole-3-acetic acid potentiates pancreatic cancer chemotherapy in mice via neutrophil-dependent myeloperoxidase oxidation, generating antiproliferative metabolites [49].

### 4.2. The Addition of 25(OH)D3 Can Affect the Inflammatory Response of Yellow Catfish

Activated inflammatory cells co-secrete both pro- and anti-inflammatory cytokines. The interaction between these two groups constructs an intricate network structure, within which dynamic balance plays a pivotal role in inflammation regulation [50]. The former category includes *IL-2*, *TNF-α*, *IL-6*, *IL-8*, interferon-γ (*IFN-γ*), and *IL-12*, all of which enhance inflammatory responses; the latter encompasses *IL-4*, *IL-13*, *IL-10*, *TGF-β*, and interleukin-1 receptor antagonist (*IL-1Ra*), whose primary function is to suppress inflammation [51]. When pathogenic microorganisms invade the host, pro-inflammatory cytokines activate both innate and adaptive immune mechanisms, thereby effectively eliminating the invaders. Following pathogen clearance, anti-inflammatory cytokines function to resolve inflammation and restore the host to normal immunological and physiological homeostasis. Previous research demonstrated that increasing dietary VD3 content improved immune function in yellow catfish by down-regulating *IFN-β* and pro-inflammatory cytokines (*TNF-α*, *IL-1β*, *IL-6*, *IL-8*) while up-regulating the anti-inflammatory cytokine *IL-10* [52]. qRT-PCR revealed decreased intestinal pro-inflammatory cytokines *(IL-1β*, *TNF-α*) versus controls while monitoring *TGF-β* expression, indicating that 25(OH)D3 may inhibit intestinal inflammation in yellow catfish through TGF-β modulation.

Quinoline-4-carboxylic acid and L-pyroglutamic acid may inhibit NF-κB through structural analogs, consistent with macrophage studies [53]. Quinoline-4-carboxylic acid exerted impressively appreciable anti-inflammation affinities without related cytotoxicities in inflamed macrophages [54]. L-pyroglutamic acid from Djulis extract inhibits NF-κB by targeting the RELA subunit [53], while benzoic acid modulates chicken immunity by up-regulating IFN-γ and down-regulating *IL-10/TLR4* [55]. In addition, the up-regulated DEGs in the 25(OH)D3 and control groups was enriched in the entry of organic acid metabolic process (GO:0006082), many of which were associated with inflammatory responses. Inter-alpha-trypsin inhibitors *itih2* and *itih3* were likely mediators of thrombo-inflammation in preeclampsia and gestational hypertension [56]. *Itih2* was involved in the acute inflammatory response, providing candidate targets for liver hepatocellular carcinoma and cholangiocarcinoma [57]. *Eprs1* coordinates early endosomal anti-inflammatory AKT signaling [58]. On the other hand, the high expression organic acids and derivatives in 25(OH)D3 group may decrease the intestinal pH value of yellow catfish. The acidic environment selectively suppresses pathogens (*Salmonella*, *Escherichia coli*) while stimulating probiotics (*Lactobacillus*, *Bifidobacterium*). Benzoic acid significantly reduced pathogenic bacteria (*Salmonella*, *E. coli*, *Eimeria* spp.; *p* < 0.05) and tended to increase *Lactobacillus* (*p* > 0.05) [55]. P-coumaric acid increased the co-aggregation of *Lactobacillus acidophilus* LA-5 and *Lacticaseibacillus rhamnosus* GG against *E. coli*, and it decreased co-aggregation against *Staphylococcus aureus* [59].

Some differential metabolites that were significantly highly expressed in the 25(OH)D3 group were also involved in inflammatory response, such as, methyl 3-indolyacetate, (2-oxo-2, 3-dihydro-1h-indol-3-yl) acetic acid, indole, 1,2,3, 9-tetrahydro-4h-carbazol-4-one oxime, aldosterone, and L-kynurenine. Methyl-3-indolyacetate inhibits cancer cell invasion by targeting the MEK1/2-ERK1/2 signaling pathway [60]. Indole is a core anti-inflammatory agent, which is prominently shown in the drug molecule indomethacin [61]. Aldosterone stimulates proinflammatory transcription factors and the production of adhesion molecules and inflammatory cytokines and chemokines by activating mineralocorticoid receptors [62]. L-Kynurenine can expand blood vessels and regulate immune response during inflammation [63]. There is a convergence of indole/organic acid metabolites with anti-inflammatory gene expression *(itih2*, *itih3*). The significant high quantitative values of these metabolites and DEGs associated with the inflammatory response in the 25(OH)D3 group may be related to the inflammatory effects of dietary addition of 25(OH)D3.

### 4.3. The Addition of 25(OH)D3 Can Enhance the Immune Function of Yellow Catfish

Terpenoid biosynthesis up-regulation (*hmgcs1*, *fdps*, etc.) reveals a novel immunometabolic axis. Terpenoid precursors may serve as endogenous immunomodulators, analogous to insect alarm pheromones [64]. Different terpenoidal molecules have been reported to have antimicrobial, antifungal, antiviral, antiparasitic, antihyperglycemic, antiallergenic, anti-inflammatory, antispasmodic, immunomodulatory, and chemotherapeutic properties [65]. This has caused them to be of great interest to the medical field, and among the terpenoids with established medical uses are antimalarial artemisinin and the anticancer taxol [66,67,68,69]. Terpenoids exhibit diverse pharmacological properties, including antimicrobial, anti-inflammatory, immunomodulatory, and chemotherapeutic activities [65]. Clinically significant examples include artemisinin (antimalarial) and taxol (anticancer) [66,67,68,69]. However, most current studies on terpenoids focus on plant-derived terpenoids, while animal-derived terpenoids focus on insects. For instance, the terpenoid biosynthesis pathway regulates aphid alarm pheromone production [64]. Considering the evolutionary conservation of terpenoid-mediated immunomodulation, the transcriptional up-regulation of terpenoid pathways by 25(OH)D_3_ suggests its greater efficacy than VD_3_ in bolstering antimicrobial immunity in yellow catfish.

### 4.4. 25(OH)D3 May Be More Effective than VD3 in Mobilizing Lipid Metabolism of Yellow Catfish

The up-regulated DEGs in the 25(OH)D3 and VD3 groups were significantly enriched in the metabolism of lipids (steroids, fatty acids, butyrate, propionate). 25(OH)D3 has potential regulatory effects on lipid metabolism through its conversion to the active form 1,25(OH)_2_D3 [70] or through direct action [17]. Serum 25(OH)D3 levels are inversely correlated with dyslipidemia incidence, showing differential associations based on baseline lipid status [71]. Intravenous 1,25(OH)2D3 improved glucose metabolism and hypertriglyceridemia in hemodialysis patients independent of PTH suppression [72]. The 10 and 100 nM 1,25(OH)_2_D3 can inhibit lipid droplet fusion, promote lipid droplet decomposition, reduce lipid droplet volume, and inhibit lipogenesis through the PPAR-α signaling pathway [73]. 1,25(OH)2D3 reduces triacylglycerol accumulation and prevents metabolic disorders by modulating lipid/glucose metabolism [70]. In prostate cancer, it regulates lipid metabolism via miRNA-mediated PPARA signaling, influencing neutral lipid accumulation [74]. Previous studies have shown that 25(OH)D3 mobilizes lipid metabolism in the active form 1,25(OH)_2_D3 or acts directly to reduce fat synthesis. In this study, lipid metabolism-related pathways, especially the enrichment of fatty acid degradation in the 25(OH)D3 group, suggest that 25(OH)D3 may be more effective than VD3 in mobilizing lipid metabolism of yellow catfish.

From an aquaculture application perspective, while 25(OH)D3 currently carries a higher raw material cost than VD3 (approximately 3–5× price difference based on commercial quotes), two factors may justify its adoption: (1) the demonstrated higher bioavailability may allow for reduced inclusion doses while maintaining efficacy; (2) the improved antioxidant and anti-inflammatory effects could decrease mortality rates and medical costs under intensive farming. Current limitations for widespread adoption include regulatory approval status in some regions and limited large-scale feeding trial data. Future research should conduct comprehensive cost–benefit analyses under commercial production conditions to validate its economic viability. While this study demonstrates the superior efficacy of dietary 25(OH)D3 over VD3 in enhancing antioxidant–inflammatory responses in yellow catfish, several limitations should be noted. First, the study tested only one dosage (2500 IU/kg) of both VD3 and 25(OH)D3, leaving the optimal supplementation levels undefined. Second, the 8-week intervention period precludes assessment of the long-term metabolic effects or potential toxicity. Third, the focus on intestinal and head kidney responses may overlook tissue-specific variations in nutrient metabolism. Additionally, the absence of a dose–response design limits translational applicability for aquaculture practices. Future studies should validate these findings across multiple doses, durations, and physiological stages to establish practical dietary recommendations.

## 5. Conclusions

This study demonstrates that dietary 25(OH)D3 supplementation significantly enhances antioxidant capacity and modulates inflammatory responses in *Pelteobagrus fulvidraco* compared to both control and VD3 treatments. Compared to the control group, all experimental groups exhibited elevated activities of antioxidase (SOD, T-AOC, CAT) and anti-inflammatory cytokines (*TGF-β)*, while lipid peroxidation product (MDA) and pro-inflammatory cytokines (*IL-1β*, *TNF-α)* were reduced. The activities of T-AOC and CAT in the 25(OH)D3 group were higher and *TNF-α* was lower compared to in the VD3 group. The quantitative values of many organic acids and indole compounds in the 25(OH)D3 group were higher than those in the control group and the VD3 group. KEGG enrichment analysis revealed significantly high expression of metabolites, such as aldosterone, L-kynourine, indole-3-acetic acid, and indole, in the 25(OH)D3 group. Furthermore, in the 25(OH)D3 and VD3 groups, the up-regulated DEGs GO function were significantly enriched in processes related to antioxidant defense. The enrichment of organic acid metabolic processes correlated with metabolomic profiles. These findings have practical significance for aquaculture nutrition, indicating that dietary 25(OH)D3 supplementation at 2500 IU/kg can effectively enhance antioxidant defenses in farmed yellow catfish.

## Figures and Tables

**Table 1:** Effects of 25(OH)D3 replacement of VD3 on antioxidant enzyme activities in the intestine.

Enzyme	Control	VD3	25(OH)D3
SOD U/mgprot	52.72 ± 4.06 ^a^	58.15 ± 3.63 ^b^	60.24 ± 4.53 ^b^
T-AOC U/mgprot	6.40 ± 0.53 ^a^	8.12 ± 0.67 ^b^	12.24 ± 1.34 ^c^
CAT U/mgprot	34.51 ± 5.49 ^a^	42.26 ± 1.85 ^b^	46.12 ± 2.74 ^c^
MDA U/mgprot	11.64 ± 0.67 ^b^	8.15 ± 0.46 ^a^	9.98 ± 0.72 ^ba^

Note: Different superscript letters within each row indicate significant differences (*p* < 0.05).

**Table 2:** Summary table of transcriptome sample data quality.

Sample	RawBase(G)	CleanBase(G)	Effective (%)	Error (%)	Q20 ^1^ (%)	Q30 ^2^ (%)	GC (%)
control_1	6.2	5.97	90.37	0.03	97.77	93.75	45.66
control_2	6.62	6.37	95.58	0.03	97.81	93.86	45.9
control_3	6.57	6.31	95.05	0.03	97.9	94.08	45.69
control_4	6.64	6.41	94.85	0.03	97.83	93.96	45.74
control_5	6.15	5.92	94.73	0.03	97.95	94.14	45.3
VD3_1	6.15	5.97	93.75	0.02	97.97	94.24	44.63
VD3_2	6.38	6.22	94.57	0.02	97.99	94.22	45.64
VD3_3	6.08	5.95	95.55	0.03	97.53	93.09	44.37
VD3_4	6.7	6.49	95.04	0.03	97.9	94.16	44.82
VD3_5	6.15	5.92	96.51	0.03	97.72	93.45	45.51
25(OH)D3_1	6.32	6.19	93.08	0.03	97.66	93.38	45.2
25(OH)D3_2	6.31	6.12	95.26	0.02	97.98	94.24	45.3
25(OH)D3_3	6.41	6.18	96.30	0.03	97.55	93.21	45.21
25(OH)D3_4	6.59	6.35	95.75	0.03	97.92	94.16	45.63
25(OH)D3_5	5.91	5.69	96.15	0.03	97.86	94.06	46.11

^1,2^ Q20 and Q30 represent the percentages of bases with Phred scores >20 and >30, respectively.

**Table 3:** The gene information of up-regulated DEGs in the 25(OH)D3 and VD3 groups.

Term	Name	25(OH)D3	VD3	*p* Value	Gene_Description
antioxidant defense	*mthfr*	232	102	2 × 10^−6^	methylenetetrahydrofolate reductase (NAD(P)H)
	*sqlea*	326	61	0.002	squalene epoxidase a
	*nsdhl*	394	230	0.003	NAD(P) dependent steroid dehydrogenase-like
	*impdh2*	489	328	0.004	IMP (inosine 5′-monophosphate) dehydrogenase 2
	*gcdhb*	2176	1659	0.005	glutaryl-CoA dehydrogenase b
	*pdpr*	633	459	0.008	pyruvate dehydrogenase phosphatase regulatory subunit
	*acadvl*	540	400	0.009	acyl-CoA dehydrogenase very long chain
	*fasn*	488	274	0.012	fatty acid synthase
	*pgd*	275	203	0.017	phosphogluconate dehydrogenase
	*mpx*	32	10	0.018	myeloid-specific peroxidase
	*prdx2*	267	196	0.022	peroxiredoxin 2
	*pam*	1590	1307	0.026	peptidylglycine alpha-amidating monooxygenase
	*msmo1*	314	112	0.026	methylsterol monooxygenase 1
	*etfdh*	906	673	0.027	electron transfer flavoprotein dehydrogenase
	*acadm*	3743	2801	0.027	acyl-CoA dehydrogenase medium chain
	*ldhba*	17,420	14,144	0.033	lactate dehydrogenase Ba
	*acads*	900	753	0.033	acyl-CoA dehydrogenase short chain
	*aldh1l2*	47	25	0.038	aldehyde dehydrogenase 1 family
	*hmgcra*	1952	746	0.040	3-hydroxy-3-methylglutaryl-CoA reductase a
	*acox1*	4158	3474	0.041	acyl-CoA oxidase 1
	*gys2*	4734	3929	0.042	glycogen synthase 2
	*hadhab*	1336	1015	0.044	hydroxyacyl-CoA dehydrogenase trifunctional multienzyme complex subunit alpha b
	*nox5*	28	16	0.045	NADPH oxidase%2C EF-hand calcium binding domain 5
	*aldh4a1*	1361	1107	0.047	aldehyde dehydrogenase 4 family%2C member A1
terpenoid backbone biosynthesis	*mvda*	69	24	2 × 10^−^	mevalonate (diphospho) decarboxylase a
	*fdps*	431	126	2 × 10^−3^	farnesyl diphosphate synthase
	*hmgcs1*	1522	664	0.001	3-hydroxy-3-methylglutaryl-CoA synthase 1 (soluble)
	*idi1*	262	142	0.003	isopentenyl-diphosphate delta isomerase 1
	*nus1*	47	26	0.010	NUS1 dehydrodolichyl diphosphate synthase subunit
	*acat2*	263	180	0.021	acetyl-CoA acetyltransferase 2
	*hmgcra*	1952	746	0.040	3-hydroxy-3-methylglutaryl-CoA reductase a

**Table 4:** The gene information of up-regulated DEGs in the 25(OH)D3 and control groups.

Term	Name	25(OH)D3	Control	*p* Value	Gene_Description
organic acid metabolic	*ehhadh*	5684	3761	0.0116	enoyl-CoA%2C hydratase/3-hydroxyacyl CoA dehydrogenase
	*itih3*	97,529	72,747	0.0161	inter-alpha-trypsin inhibitor heavy chain 3
	*eprs1*	8679	7088	0.0179	glutamyl-prolyl-tRNA synthetase 1
	*itih2*	51,007	42,246	0.0206	inter-alpha-trypsin inhibitor heavy chain 2
	*hdc*	25	11	0.0259	histidine decarboxylase
	*dars1*	1846	1272	0.0324	aspartyl-tRNA synthetase 1
	*mthfr*	249	136	0.0413	methylenetetrahydrofolate reductase (NAD(P)H)
	*pklr*	3086	2316	0.0435	pyruvate kinase L/R
	*yars2*	57	39	0.0462	tyrosyl-tRNA synthetase 2%2C mitochondrial

## Data Availability

All data generated and analyzed during this study are included in the published article.

## Supplementary Materials

The following supporting information can be downloaded at https://www.mdpi.com/article/10.3390/biology14080967/s1: Table S1: Feed nutritional components (%, air-dried matter); Table S2: Primer Sequences; Table S3: The differentially expressed POS and four NEG metabolites between the control group and the VD3 group; Table S4: The differentially expressed POS and four NEG metabolites between the control group and the 25(OH)D3 group; Table S5: The differentially expressed POS and four NEG metabolites between the VD3 group and the 25(OH)D3 group; Table S6: KEGG enrichment of differentially expressed POS and four NEG metabolites between the control group and the 25(OH)D3 group; Table S7: KEGG enrichment of differentially expressed POS and four NEG metabolites between the VD3 group and the 25(OH)D3 group.
