# Supplementary material for: The Effects of Dietary Supplementation with 25-Hydroxyvitamin D3 on the Antioxidant Capacity and Inflammatory Responses of Pelteobagrus fulvidraco
Source: Biology (Basel). 2025 Aug 1;14(8):967. doi: 10.3390/biology14080967 (PMC12383693; doi:10.3390/biology14080967)
Supplement: Supplementary file 1 [file biology-14-00967-s001.zip › biology-3753430-supplementary.pdf]

Table S1 Feed nutritional components (%, air-dried matter)

Raw materials	Content (%)
Fish meal	28
Soybean meal	27
Wheat flour	28
Soybean oil	6.5
Chicken powder	8
Soy lecithin	0.2
Mineral premix	0.1
Vitamin premix	0.1
Acetylcholine	0.1
Calcium dihydrogen phosphate	1
Crystalline cellulose	1
Approximate nutritional components (%, air-dried matter)	-
Crude protein	43.36
Crude lipid	8.67
Crude ash	9.35

Note: Mineral premix (mg/kg): Mg 26 g; Fe 8 g; Mn 2 g; I 500 mg; Cu 1 g; Zn 5 g; Se 35 mg; Co 100 mg. Vitamin premix (mg/kg): VA 200000 IU; Vitamin C 11000 mg; VE 4500 mg; Vitamin K3 480 mg; Vitamin B1 500 mg; Vitamin B2 750 mg; Vitamin B6 650 mg; Vitamin B12 2 mg; Inositol 3000 mg; Nicotinamide 3200 mg; D-calcium pantothenate 1500 mg; Folic acid 130 mg; D-biotin 15 mg.

Table S2 Primer Sequences

Genes	Sequence（5'-3'）	Gene number
IL-1β	F：CTGTGTGTTTGGGGATTGTG	MF770571
	R：GGGTATTTCACCGACTCGAA	
IL-8	F：GACTGCGATGCTTTGTGAAG	KY218792
	R：TCAGGCAGACCTTCATTCCT	
TNF-α	F：TCTGCTTCACCATCTTCGTG	XM_027165847
	R：GGCACCAGCTTCTTGACTTC	
TGF-β	F：ACAGGTCCAAAGGTTGATGG	XM_027154377
	R：CACATCCACAAGAAGCTGGA	
β-actin	F：GATTCGCTGGAGATGATGCT	KM673246.1
	R：CGTGCTCAATGGGGTACTTC	

Table S3 The differentially expressed POS and four NEG metabolites between the control group and the VD3 group

	Name	Formula	RT [min]	m/z	log2FC	Pvalue	Up.Down
POS	Proline-hydroxyproline	C10 H16 N2 O4	5.12	212.09	-1.08	0.02	down
	PS (18:0/18:1)	C42 H80 N O10 P	11.35	790.56	-5.14	0.02	down
	PQH	C16 H24 N6 O5	4.94	381.19	-1.15	0.04	down
	Propionylcarnitine	C10 H19 N O4	2.81	201.11	-2.21	0.04	down
	1,2,3,9-tetrahydro-4H-carbazol-4-one oxime	C12 H12 N2 O	6.22	201.10	2.12	0.04	up
	3-(4-morpholinophenyl)-2-(4-nitrophenyl)acrylonitrile	C19 H17 N3 O3	5.47	336.13	-0.88	0.05	down
NEG	Methyl 3-indolyacetate	C11 H11 N O2	6.00	188.07	-1.62	0.01	down
	Dodecanedioic acid	C12 H22 O4	6.56	229.15	0.39	0.01	up
	Pentadecanoic Acid	C15 H30 O2	7.07	287.22	0.86	0.02	up
	Indole-3-propionic acid	C11 H11 N O2	5.65	188.07	-0.65	0.04	down

Note: POS: Positive ion mode; NEG: Negative ion mode; RT [min], retention time; m/z, mass-to-charge ratio; log2FC, compare the log2 values of the multiple of difference; Pvalue, compare the significance of p-value.

Table S4 The differentially expressed POS and four NEG metabolites between the control group and the 25(OH)D3 group

	Name	Formula	RT [min]	m/z	log2FC	Pvalue	Up.Down
POS	L-Glutamic acid	C5 H9 N O4	1.27	148.06	-0.43	0.01	down
	7-Hydroxy-3,4-dihydrocarbostyril	C9 H9 N O2	4.78	164.07	-0.62	0.02	down
	Riboflavin-5-phosphate	C17 H21 N4 O9 P	5.35	457.11	1.86	0.02	up
	WKK	C23 H36 N6 O4	4.76	214.12	1.87	0.02	up
	9H-Pyrido (3,4-B) indole	C11 H8 N2	5.25	169.08	-1.53	0.02	down
	1,2-dihydroxyheptadec-16-yn-4-yl acetate	C19 H34 O4	7.37	309.24	0.82	0.02	up
	1-(4-methylphenyl)-3,5-diphenylpent-2-ene-1,5-dione	C24 H20 O2	5.78	323.14	-2.54	0.02	down
	Indole-3-acetic acid	C10 H9 N O2	5.03	176.07	-1.57	0.03	down
	N-(2-hydroxyphenyl)acetamide	C8 H9 N O2	5.66	174.05	-0.62	0.03	down
	L-Pyroglutamic acid	C5 H7 N O3	1.52	130.05	-0.28	0.03	down
	Indole	C8 H7 N	4.83	118.06	-0.36	0.04	down
	Methyl indole-3-acetate	C11 H11 N O2	5.69	190.09	-0.89	0.04	down
	Isoquinoline	C9 H7 N	5.14	130.06	-0.48	0.04	down
	Glucose 1-phosphate	C6 H13 O9 P	1.31	261.04	0.77	0.04	up
	1,2,3,9-tetrahydro-4H-carbazol-4-one oxime	C12 H12 N2 O	6.22	201.10	1.92	0.05	up
	5-Hydroxyindole	C8 H7 N O	5.14	134.06	-0.38	0.05	down

	L-Kynurenine	C10 H12 N2 O3	4.62	209.09	-1.32	0.05	down
	Coumarin	C9 H6 O2	2.28	147.04	-0.35	0.05	down
NEG	Allantoin	C4 H6 N4 O3	1.32	157.04	0.45	0.02	up
	3-(2-Naphthyl)-D-Alanine	C13 H13 N O2	6.42	214.09	-2.11	0.02	down
	(2-oxo-2,3-dihydro-1H-indol-3-yl)acetic acid	C10 H9 N O3	5.53	190.05	-1.50	0.02	down
	DL-methionine sulfoxide	C5 H11 N O3 S	1.30	164.04	-1.21	0.02	down
	Quinoline-4-carboxylic acid	C10 H7 N O2	5.66	172.04	-1.01	0.02	down
	Homotaurine	C3 H9 N O3 S	1.27	138.02	-0.38	0.03	down
	N-Carbamyl-L-glutamicacid	C6 H10 N2 O5	1.26	189.05	-0.32	0.03	down
	Dl-Indole-3-lactic acid	C11 H11 N O3	5.66	204.07	-0.83	0.03	down
	Benzoic acid	C7 H6 O2	5.47	121.03	-1.09	0.03	down
	α-D-Mannose 1-phosphate	C6 H13 O9 P	1.30	259.02	0.71	0.03	up
	1-benzyl-3-(2-methylphenyl)-3,7-dihydro-1H-purine-2,6-dione	C19 H16 N4 O2	7.49	331.12	-0.98	0.03	down
	2-(3,4-dimethoxyphenyl)ethanamine	C10 H15 N O2	6.51	180.10	-1.98	0.03	down
	LPC 20:2	C28 H54 N O7 P	9.82	592.36	-1.95	0.04	down
	Capric acid	C10 H20 O2	5.81	171.14	1.82	0.04	up
	Terephthalic Acid	C8 H6 O4	2.94	165.02	-1.36	0.04	down
	Aldosterone	C21 H28 O5	1.10	359.18	-0.63	0.05	down
	Maltotriose	C18 H32 O16	1.39	539.14	-1.45	0.05	down
	3-Coumaric acid	C9 H8 O3	2.25	163.04	-0.40	0.05	down
	11(Z),14(Z)-Eicosadienoic Acid	C20 H36 O2	10.74	307.27	-1.64	0.05	down

Table S5 The differentially expressed POS and four NEG metabolites between the VD3 group and the 25(OH)D3 group

	Name	Formula	RT [min]	m/z	log2FC	Pvalue	Up.Down
POS	7-Hydroxy-3,4-dihydrocarbostyril	C9 H9 N O2	4.78	164.07	-0.90	0.01	down
	8-Aminooctanoic acid	C8 H17 N O2	1.43	160.13	0.49	0.01	up
	Ethylmalonate	C5 H8 O4	6.81	133.05	1.63	0.01	up
	Thymine	C5 H6 N2 O2	2.97	127.05	1.09	0.01	up
	8,8-dimethyl-2-phenyl-4H,8H-pyrano[2,3-h]chromen-4-one	C20 H16 O3	5.68	305.12	-1.11	0.02	down
	9H-Pyrido (3,4-B) indole	C11 H8 N2	5.25	169.08	-1.93	0.02	down
	1-oxo-2,3-dihydro-1H-inden-4-yl benzoate	C16 H12 O3	6.14	270.11	-2.12	0.02	down
	Enrofloxacin	C19 H22 F N3 O3	5.33	360.18	1.92	0.03	up
	PS (18:0/18:1)	C42 H80 N O10 P	11.35	790.56	3.47	0.03	up
	Riboflavin-5-phosphate	C17 H21 N4 O9 P	5.35	457.11	1.28	0.03	up
	DL-Arginine	C6 H14 N4 O2	1.19	175.12	-0.36	0.03	down
	3-Indoleacetonitrile	C10 H8 N2	5.14	140.05	-0.48	0.03	down
	Indole	C8 H7 N	4.83	118.06	-0.36	0.03	down

	o-Cresol	C7 H8 O	4.83	109.06	-0.32	0.03	down
	3-hydroxy-1,5-diphenylpentan-1-one	C17 H18 O2	5.58	277.12	-0.72	0.04	down
	L-(+)-Citrulline	C6 H13 N3 O3	1.27	176.10	0.69	0.04	up
	R-1 Methanandamide phosphate	C23 H40 N O5 P	4.88	464.25	1.16	0.04	up
	1-(4-methylphenyl)-3,5-diphenylpent-2-ene-1,5-dione	C24 H20 O2	5.78	323.14	-1.97	0.04	down
	PQH	C16 H24 N6 O5	4.94	381.19	1.47	0.04	up
	L-Kynurenine	C10 H12 N2 O3	4.62	209.09	-1.32	0.04	down
	ANH	C13 H20 N6 O5	6.81	341.16	2.80	0.05	up
NEG	IDP	C10 H14 N4 O11 P2	1.12	427.01	-0.41	0.01	down
	3-Coumaric acid	C9 H8 O3	2.25	163.04	-0.58	0.02	down
	GDP	C10 H15 N5 O11 P2	1.95	442.02	1.00	0.02	up
	Methyl 3-indolyacetate	C11 H11 N O2	6.00	188.07	1.13	0.03	up
	Allantoin	C4 H6 N4 O3	1.32	157.04	0.89	0.03	up
	(2-oxo-2,3-dihydro-1H-indol-3-yl)acetic acid	C10 H9 N O3	5.53	190.05	-1.30	0.04	down
	(±)8-HEPE	C20 H30 O3	7.78	317.21	0.63	0.04	up
	2-Isopropylmalate	C7 H12 O5	4.82	175.06	-0.75	0.04	down
	Aldosterone	C21 H28 O5	1.10	359.18	-0.71	0.04	down
	Homovanillic acid	C9 H10 O4	5.61	181.05	2.08	0.05	up
	4-Methylhippuric acid	C10 H11 N O3	5.62	192.07	-1.24	0.05	down

Table S6 KEGG enrichment of differentially expressed POS and four NEG metabolites between the control group and the 25(OH)D3 group

	MapID	MapTitle	Pvalue	MetaIDs
POS	map00380	Tryptophan metabolism	0.004	L-Kynurenine; Indole-3-acetic acid; Indole
	map00480	Glutathione metabolism	0.01	L-Pyroglutamic acid; L-Glutamic acid
	map05143	African trypanosomiasis	0.05	L-Kynurenine
	map00190	Oxidative phosphorylation	0.11	Riboflavin-5-phosphate
	map00650	Butanoate metabolism	0.11	L-Glutamic acid
	map00660	C5-Branched dibasic acid metabolism	0.11	L-Glutamic acid
	map00740	Riboflavin metabolism	0.11	Riboflavin-5-phosphate
	map00220	Arginine biosynthesis	0.15	L-Glutamic acid
	map00250	Alanine, aspartate and glutamate metabolism	0.15	L-Glutamic acid
	map00400	Phenylalanine, tyrosine and tryptophan biosynthesis	0.15	Indole
	map00430	Taurine and hypotaurine metabolism	0.15	L-Glutamic acid
	map00471	D-Glutamine and D-glutamate metabolism	0.15	L-Glutamic acid
	map00524	Neomycin, kanamycin and gentamicin biosynthesis	0.15	L-Glutamic acid
	map00630	Glyoxylate and dicarboxylate metabolism	0.15	L-Glutamic acid
	map00910	Nitrogen metabolism	0.15	L-Glutamic acid
	map00860	Porphyrin and chlorophyll metabolism	0.20	L-Glutamic acid
	map04977	Vitamin digestion and absorption	0.29	Riboflavin-5-phosphate

	map00340	Histidine metabolism	0.33	L-Glutamic acid
	map00970	Aminoacyl-tRNA biosynthesis	0.37	L-Glutamic acid
	map00330	Arginine and proline metabolism	0.41	L-Glutamic acid
	map04974	Protein digestion and absorption	0.41	Indole
	map01100	Metabolic pathways	0.66	L-Kynurenine; Indole-3-acetic acid; L-Glutamic acid; Indole; Riboflavin-5-phosphate
NEG	map04973	Carbohydrate digestion and absorption	0.05	Maltotriose
	map00051	Fructose and mannose metabolism	0.09	α-D-Mannose 1-phosphate
	map04960	Aldosterone-regulated sodium reabsorption	0.09	Aldosterone
	map04925	Aldosterone synthesis and secretion	0.21	Aldosterone
	map00520	Amino sugar and nucleotide sugar metabolism	0.28	α-D-Mannose 1-phosphate
	map02010	ABC transporters	0.32	Maltotriose
	map00140	Steroid hormone biosynthesis	0.35	Aldosterone
	map00360	Phenylalanine metabolism	0.38	Benzoic acid
	map01100	Metabolic pathways	1.00	Aldosterone; Benzoic acid; α-D-Mannose 1-phosphate; Terephthalic Acid

Table S7 KEGG enrichment of differentially expressed POS and four NEG metabolites between the VD3 group and the 25(OH)D3 group

	MapID	MapTitle	Pvalue	MetaIDs
POS	map00380	Tryptophan metabolism	0.004	L-Kynurenine; 3-Indoleacetonitrile; Indole
	map05143	African trypanosomiasis	0.05	L-Kynurenine
	map00190	Oxidative phosphorylation	0.11	Riboflavin-5-phosphate
	map00740	Riboflavin metabolism	0.11	Riboflavin-5-phosphate
	map00400	Phenylalanine, tyrosine and tryptophan biosynthesis	0.15	Indole
	map04977	Vitamin digestion and absorption	0.29	Riboflavin-5-phosphate
	map04974	Protein digestion and absorption	0.41	Indole
	map00240	Pyrimidine metabolism	0.54	Thymine
	map01100	Metabolic pathways	0.66	L-Kynurenine; Thymine; o-Cresol; Indole; Riboflavin-5-phosphate
NEG	map03013	RNA transport	0.04	GDP
	map04014	Ras signaling pathway	0.04	GDP
	map04015	Rap1 signaling pathway	0.04	GDP
	map04144	Endocytosis	0.04	GDP
	map00230	Purine metabolism	0.05	GDP; IDP
	map04728	Dopaminergic synapse	0.07	Homovanillic acid
	map04960	Aldosterone-regulated sodium reabsorption	0.07	Aldosterone
	map00350	Tyrosine metabolism	0.17	Homovanillic acid
	map04925	Aldosterone synthesis and secretion	0.17	Aldosterone
	map00140	Steroid hormone biosynthesis	0.29	Aldosterone
